# Reproductive outcome after hysteroscopic septoplasty in patients with septate uterus - a retrospective cohort study and systematic review of the literature

**DOI:** 10.1186/1477-7827-8-52

**Published:** 2010-05-21

**Authors:** Kazem Nouri, Johannes Ott, Johannes C Huber, Eva-Maria Fischer, Lucija Stögbauer, Clemens B Tempfer

**Affiliations:** 1 Department of Gynecologic Endocrinology and Reproductive Medicine, Medical University of Vienna, Vienna, Austria; 2 Department of Gynecology and Gynecologic Oncology, Medical University of Vienna, Vienna, Austria

## Abstract

**Background:**

Septate uterus, one of the most common forms of congenital uterine malformations, negatively affects female reproductive health.

**Methods:**

In a retrospective cohort study, we evaluated the reproductive outcome after hysteroscopic septoplasty in 64 women with septate uterus and primary or secondary infertility. We performed a systematic review of studies evaluating the reproductive outcome after hysteroscopic septoplasty.

**Results:**

Sixty-four women underwent hysteroscopic septoplasty. In 2/64 (3%) women, intraoperative uterine perforation occurred. Complete follow-up was available for 49/64 (76%) patients. Mean follow-up time was 68.6 +/- 5.2 months. The overall pregnancy rate after hysteroscopic septoplasty was 69% (34/49). The overall life birth rate (LBR) was 49% (24/49). The mean time interval between surgery and the first life birth was 35.8 +/- 22.5 months. Including our own data, we identified 18 studies investigating the effect of septoplasty on reproductive outcome in 1501 women. A pooled analysis demonstrated that hysteroscopic septoplasty resulted in an overall pregnancy rate of 60% (892/1501) and a LBR of 45% (686/1501). The overall rate of intra- and postoperative complications was 1.7% (23/1324) and the overall rate of re-hysteroscopy was 6% (79/1324).

**Conclusions:**

In women with septate uterus and a history of infertility, hysteroscopic septoplasty is a safe and effective procedure resulting in a pregnancy rate of 60% and a LBR of 45%.

## Background

Septate uterus, i.e. an incompletely septated uterus or uterus subseptus, is one of the most common forms of congenital uterine malformations [1]. The incidence of congenital uterine malformations has been reported to be as high as 3-4% in the general female population [2,3] and to be significantly higher in patients with infertility and recurrent pregnancy loss [3,4].

Septate uterus results from incomplete resorption of the paramesonephric muellerian ducts during the first trimester of pregnancy. The absorption of the septum normally initiates at the level of the uterine cervix and continues upwards in the direction of the uterine fundus. Depending on the size of the septum, the uterine cavity may be affected only partially, as in case of an incomplete septate uterus, or it may be divided into two separate components including two cervices and eventually a vaginal septum, as in case of a complete septate uterus.

A uterine septum affects female reproductive health in three ways: (i) obstetric complications, (ii) recurrent miscarriages, and (iii) infertility [5]. Although clinical studies consistently demonstrate a poorer obstetric outcome in patients with septate uterus compared to women without uterine anomalies [5,6], literature on septate uterus as the primary cause of female infertility is controversial. Fedele et al., for example, were the first to postulate that septate uterus may influence fertility by hindering embryo implantation [7]. This hypothesis was based on histological samples obtained during operative hysteroscopy demonstrating the following ultra-structural alterations in the septal endometrium compared to endometrium of the lateral uterine wall: (i) a reduced number of glandular ostia, (ii) irregularly distributed ciliated cells with incomplete ciliogenesis, and (iii) a reduction of the ciliated to non-ciliated cell ratio. These factors are believed to cause the poor response to estrogens in the septal mucosa, since normal serum estrogens levels were found in all patients. Others demonstrated inadequate uterine vascularisation leading to subsequent abnormal placentation in women with a septate uterus [8]. Moreover, clinical studies in women with septate uterus reported an increased content of muscle tissue as well as an increased and uncoordinated contractility of the uterine septum [9].

On the other hand, Sparac found no differences in vascularisation assessed by transvaginal colour Doppler ultrasound comparing endometrium and uterine septum [9]. In another study, histological examination of uterine septum biopsies from three different sites of the septum, namely the basis, the midpoint, and the tip, demonstrated no differences in terms of muscle tissue and vessel density compared to biopsies from the left posterior aspect of the uterus [10].

Available literature on reproductive outcome after uterine septoplasty is inconsistent. For example, pregnancy rates ranging from 39% [11] to 81% [12] and life birth rates ranging from 26% [13] to 73% [14] have been reported. Most of these studies were retrospective. To our best knowledge, no prospective randomised trial comparing septoplasty to no intervention has been published and is, for ethical reasons, unikely to be conducted in the future.

The aim of our study was to examine the safety and efficacy of hysteroscopic correction of septate uterus by hysteroscopic uterine septoplasty using a retrospective series of affected women. Furthermore, we conducted a systematic review and pooled analysis of studies reporting on reproductive outcome, complications, and re-operation rates after this procedure.

## Methods

### Patients

We retrospectively evaluated all patients having undergone hysteroscopic resection of a uterine septum at our department from 1997 to 2006. Primary outcome parameter was obstetric outcome (number of pregnancies, number of life births). As a second outcome parameter we evaluated the number and type of intra- and postoperative complications in order to gain information on the safety of the procedure.

A total of 64 women were enrolled in the study. Fourteen patients (22%) presented with primary infertility, defined as the inability to conceive after 12 months of contraceptive-free intercourse. Fifty patients (78%) presented with secondary infertility, defined as the inability to conceive after 12 months of contraceptive-free intercourse after having already conceived at least once. Twenty-two of 50 patients with secondary infertility (44%) have been diagnosed with recurrent abortion, defined as three or more consecutive spontaneous pregnancy losses before 20 weeks gestation. Written informed consent was obtained from patients for publication of the data presented. Copies of the written consents are available for review by the Editor-in-Chief of this journal.

### Surgical technique

All operations were performed as in-patient surgery. We neither used any form of long-term pre-treatment with danazol or gonadotropin releasing hormone (GnRH) agonists, nor pre-operative intravaginal administration of misoprostol in contrast to other investigators [15]. Uterine septoplasty was performed under general anaesthesia by two surgeons combining hysteroscopy and diagnostic laparoscopy. All procedures were performed in the follicular phase of the menstrual cycle, usually within seven days after the end of menstruation. In 38 women patency of the fallopian tubes was tested by chromo-pertubation with Lipiodol (Lipiodol Ultrafluid Ampul™, Guerbet, Austria) which was performed after septoplasty.

After cervical dilation with Hegar's dilators up to a width of 10 mm, an operative hysteroscope (Storz 10 mm fibreoptic resectoscope; Storz GmbH, Germany) was inserted. The uterine cavity was distended with a 1.5% glycine solution with a continuous irrigating flow. The hysteroscope's hook was placed in contact with the septum and an incision was made perpendicular to the septum. All operations were performed with monopolar electrosurgery, no scissors or lasers were used. The aim of the procedure was to accomplish a triangular and symmetric uterine cavity, which was achieved in all of the patients Uterine septoplasty was performed under laparoscopic supervision.

### Follow up

All patients were sent a questionnaire per mail. The following questions were evaluated: whether the patient tried to get pregnant after septoplasty; the number of pregnancies achieved; the number of miscarriages, extrauterine pregnancies, and spontaneous abortions, and the number of life births including information on gestational week and delivery mode; and whether patients had undergone any form of assisted reproductive techniques. All patients were asked to return the completed questionnaire and, in case that they had delivered in another hospital, to enclose a copy of the hospital delivery report or another document proving the life birth of a child.

### Statistical analysis

Variables are described by frequencies and mean ± standard deviation (SD) of the mean. Differences between groups were analyzed by Chi square and Fisher's exact test. A p-value < 0.05 was considered statistically significant. Multiple comparisons were corrected by Bonferroni's correction. Statistical analysis was performed in SPSS 15.0.1 for Windows (SPSS Inc, 1989-2006).

### Systematic literature review

We searched Medline and EMBASE and the Cochrane controlled trials register (search date: 20.12.2009; search terms: uterus subseptus, septate uterus, infertility, hysteroscopy, septoplasty) to identify cohort studies, systematic reviews, and meta-analyses evaluating the reproductive outcome after hysteroscopic septoplasty in women diagnosed with septate uterus. Studies were included if they were published as complete reports in English. Bibliographies of studies were searched for relevant citations. Multiple studies describing the same study population were included once. In this case, the original publication was used, i.e. the one with the earliest date of publication. Two authors assessed the eligibility of the studies and extracted data (KN and JO). Missing information and additional trials were not sought from authors. Data on pregnancy, life birth rates, complications, and re-hysteroscopy rates were extracted, pooled, and re-analyzed.

## Results

### Surgical procedure

Fifty-seven of 64 patients (89%) underwent uterine septoplasty as described above including diagnostic laparoscopy and operative hysteroscopy. The mean age of the patients was 30.3 ± 5.7 years, the mean body mass index was 23.9 ± 2.7, and the mean duration of infertility was 4.5 ± 2.7 years. In 4/64 patients (6%) only operative hysteroscopy for dissection of the uterine septum was performed, whereas 3 patients (4%) underwent an additional curettage. Sixty-two patients had uneventful intraoperative and postoperative courses. However, in 2/64 (3%) patients, uterine perforation occurred. In both cases no adjacent organs were injured and the complication was managed by bipolar coagulation. No sutures were applied. No postoperative episode of fever was noted.

### Re-septoplasty

In 8/64 (13%) patients a second intervention was necessary since the uterine septum had not been completely removed during the first operation. The re-operation was done after a mean of 14.4 ± 10.7 months. Re-septoplasty was more often necessary in patients who had undergone hysteroscopy only than in those who had undergone combined hysteroscopy and laparoscopy (2/4 [50% vs. 6/45 [11%]; p = 0.07).

### Obstetric outcome

Complete follow-up was available for 49/64 (77%) patients. Mean follow-up time was 68.6 ± 25.2 months. All of the patients tried to become pregnant after septoplasty. The overall pregnancy rate after septoplasty was 69% (34/49). Eleven patients reported more than one pregnancy. Specifically, six women had two pregnancies, two women had three pregnancies, two women had four pregnancies, and one woman had six pregnancies, respectively. Thus, we registered a total of 49 pregnancies. Sixteen pregnancies had resulted from in vitro fertilization (33%), three pregnancies from insemination (6%), and the remaining 27 pregnancies (55%) resulted from spontaneous conception.

The overall life birth rate (LBR) was 49% (24/49). All life births were confirmed by hospital birth records. Three patients reported two life births, one patient reported three life births. Table 1 shows the reproductive outcome of women with and without a history of early spontaneous miscarriages and women with and without a history of recurrent miscarriages, ie three or more miscarriages. Women with a history of recurrent miscarriages had a significantly higher pregnancy rate after uterine septoplasty compared to women without a history of recurrent miscarriages.

**Table 1:** Obstetric outcome after hysteroscopic septoplasty of women with and without a history of early miscarriage and of women with and without a history of recurrent miscarriage

	Patients with a history of early miscarriage (n = 47)	Patients without a history of early miscarriage (n = 17)	p-value
Follow-up time (months)	66.1 ± 25.6	72.6 ± 24.3	0.1
Pregnancy rate	25/37 (68%)	9/12 (75%)	0.6
Life birth rate	18/37 (49%)	6/12 (50%)	0.9
Miscarriage rate	7/37 (19%)	3/12 (25%)	0.7
	Patients with a history of recurrent miscarriage (n = 22)	Patients without a history of recurrent miscarriage (n = 42)	p-value
Follow-up time (months)	62.2 ± 23.8	69.4 ± 24.7	0.09
Pregnancy rate	16/17 (94%)	18/32 (56%)	0.04*
Life birth rate	11/17 (64%)	13/32 (40%)	0.1
Miscarriage rate	5/17 (29%)	5/32 (16%)	0.3

*after Bonferroni's correction

The mean time interval between uterine septoplasty and the first delivery was 35.8 ± 22.5 months. Out of a total of 29 life births, 9 (31%) were preterm deliveries before 37 weeks gestation. No difference regarding preterm deliveries was observed comparing patients with or without a history of recurrent miscarriages (6/21 [29%] vs. 3/8 [38%], respectively; p = 0.6).

**Figure 1:**
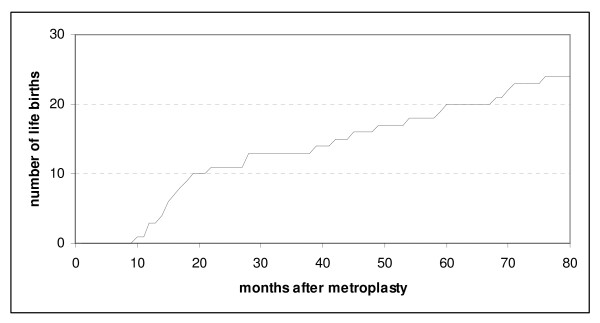
**Cumulative life birth rate after uterine septoplasty**.

Figure 1 shows a graph of the time to first life birth in women after uterine septoplasty, demonstrating a slow, but consistent increase of life births during the observation period of 80 months. Cesarean section was the delivery mode in 12/29 (41%) women. The remaining 17 babies were delivered vaginally (59%). None of the patients was diagnosed with uterine rupture in the course of pregnancy and delivery.

### Cofactors of infertility

Complete information on hormonal status, patency rates of the fallopian tubes, and the result of the partner's semen analysis was available for 21/64 (33%) women. Male factor, unilateral tubal factor, bilateral tubal factor, and hormonal pathologies, ie hyperprolactinemia, hyperandrogenemia, and thyroid dysfunction were noted in 14, 9, 3, and 11 of these women, respectively. Women with a bilateral tubal factor underwent in vitro fertilization. In 8/49 (16%) women, a change of the male partner was noted.

### Systematic review and meta-analysis

We identified 18 studies investigating the outcomes after hysteroscopic uterine septoplasty [11-28]. One study was excluded from the analysis of obstetric outcomes, because data on pregnancy rates and LBR could not be extracted [17]. Table 2 provides an overview of the individual studies including our own data and a pooled analysis of 1501 women, yielding an overall pregnancy rate of 60.1% (892/1501) and a LBR of 45.0% (686/1501) after uterine septoplasty.

**Table 2:** Literature on pregnancy rates and live birth rates after hysteroscopic uterine septoplasty in women with septate uterus

Author	Year	Patient number	Uterine mal-formation	Patient characteristics	Pregnancy rate	Live birth rate
Venturoli et al. [13]	2002	141	IUS	Infertility; RM	74/141 (52%)	56/141 (39%); 14 ongoing
Doridot et al [14]	2003	70	CUS	21 PI; 33 RM; 16 LM or PT	8/21 (38%); 13/33 (38%); 10/16 (60%)	-
Jakiel et al. [15]	2004	31	CUS	PI; RM; LM and PT	18/31 (58%)	11 (38%)
Hollett-Caines et al. [20]	2006	26	CUS	Infertility; RM	21/26 (80%)	15/26 (57%)
Pace et al. [16]	2006	70	IUS; CUS	PI; RM; PT	30/40 (75%)	25/40 (62%)
Colacurci et al. [21]	2007	135	CUS	Infertility	99/135 (73%)	82/135 (60%)
Colacurci et al. [22]	1996	69	IUS; CUS	PI; RM	46/69 (66%)	36/69 (52%); 4 ongoing
Saygili-Yimaz et al. [23]	2003	361	CUS	PI; RM	180/361 (49%)	124/361 (34%)
Pabuçcu & Gomel [24]	2004	61	CUS	PI	25/61 (41%)	18/61 (29%)
Valle RF [26]	1996	124	CUS	RM	101/124 (81%)	91/124 (73%)
Ozgur et al. [11]	2007	119	IUS	IVF	57/119 (47%)	51/119 (42%)
Marabini et al. [27]	1994	40	CUS	Infertility; RM	19/26 (73%)	13/26 (50%); 4 ongoing
Kupesic & Kurjak [28]	1998	116	CUS	Infertility	59/116 (50%)	48/116 (41%)
Porcu et al. [29]	2000	63	CUS	RM	45/56 (80%)	28/56 (50%)
Guarino et al. [31]	1989	35	CUS	PI; RM	18/35 (51%)	16/35 (45%)
Wang et al. [30]	2008	25	CUS	PI and SI; RM	13/23 (56%)	6/23 (26%); 6 ongoing
Mollo et al. [17]	2009	44	CUS	-	17/44 (38%)	15/44(34%)
Litta et al. [25]	2007	63	IUS	-	36/45 (80%)	27/45 (60%)
Nouri et al.	2010	64	IUS	PI and SI; RM	34/49 (69%)	24/49 (49%)
Total	-	1587	IUS; CUS	-	892/1501 (60%)	686/1501 (45%)

IUS = incomplete uterine septum; CUS = complete uterine septum; RM = recurrent miscarriages; PT = preterm delivery; PI = primary infertility; SI = secondary infertility; LM = late miscarriages

Details on postoperative complications and re-hysteroscopy rates are given in Table 3. For this analysis we excluded four studies [11,12,16,18] with a total of 242 patients because the complication rates were not given in these studies. The pooled analysis of 14 studies with 1324 women undergoing uterine septoplasty resulted in an overall complication rate of 1.7% (23/1324). The overall rate of re-hysteroscopy was 6.0% (79/1324). There was no statistically significant difference regarding overall PR, LBR, and complications comparing our series with the pooled data from 17 studies in the literature (data not shown).

**Table 3:** Literature on complications during and after hysteroscopic uterine septoplasty in women with septate uterus

Ref	Year	N	LSK	POT	UM	Complications				
						**P**	**PF**	**PP**	**CL**	**RP**

[13]	2002	141	-	-	IUS	-	-	-	-	-

[14]	2003	70	Yes	-	CUS	2	0	0	0	2

[15]	2004	31	-	-	CUS	-	-	-	-	-

[20]	2006	26	-	-	CUS	-	-	-	-	-

[16]	2006	70	Yes	GnRH D	IUS; CUS	0	0	0	0	0

[21]	2007	135	-	-	CUS	0	0	0	0	0

[22]	1996	69	No	GnRH	IUS; CUS	0	0	0	0	2

[23]	2003	361	No	D	CUS	10	0	0	0	49

[24]	2004	61	No	-	CUS	0	0	0	0	14

[26]	1996	124	No	Yes	CUS	0	0	0	0	4

[11]	2007	119	No	M	CUS	0	0	0	0	0

[28]	1994	40	No	D; PGE1	CUS	0	0	0	0	1

[29]	1998	116	No	-	CUS	0	0	0	0	0

[30]	2000	63	-	-	CUS	1	0	0	0	1

[31]	1989	35	No	-	CUS	0	0	0	0	0

[30]	2008	25	No	-	CUS	0	0	0	0	0

[17]	2009	44	-	-	CUS	-	-	-	-	-

[25]	2007	63	No	-	IUS	0	0	1	2	4

PS	2010	64	Yes	-	IUS	2	0	0	0	2

PA	-	1324	204 15.4%	-	IUS; CUS	15 1.1%	0	1 0.07%	2 0.1%	79 6.0%

Ref = reference; n = number of patients; Year = year of publication; UM = uterine malformation; P = perforation; PF = postoperative fever; LSK = concomitant laparoscopy; PP = pelvic pain; CL = cervical laceration; RP = re-operation; POT = pre-operative therapy; IUS = incomplete uterine septum; CUS = complete uterine septum; GnRH = gonadotropin releasing hormone agonist; D = danazol; FOS = fluid overload syndrome; M = misoprostol in 2 patients 2 hours prior to surgery; PGE1 = prostaglandin E1; IU = intrauterine adhesions; PS = present study (Nouri et al. 2010); PA = pooled analysis

## Discussion

In this retrospective cohort study of 64 women and a pooled analysis of 18 studies including 1501 women, we describe a pregnancy rate of 60% and a LBR of 45% after hysteroscopic septoplasty in women with septate uterus. We conclude that hysteroscopic septoplasty is a simple, effective, and safe procedure with an intra- and postoperative complication rate of 1.7%. Our data, therefore, support the use of this procedure and confirm a high rate of reproductive success in women with a septate uterus.

Septate uterus used to be treated by transabdominal metroplasty [29]. This surgical method has been substituted by operative hysteroscopy. The reasons were a high complication rate of abdominal metroplasty including the risk of postoperative adhesions potentially leading to infertility. Also, abdominal metroplasty was associated with an increased risk of scar rupture during pregnancy, resulting in the recommendation of cesarean section as the preferred mode of delivery [29]. Furthermore, laparotomy is associated with longer hospital stays compared to operative hysteroscopy.

The only advantage of performing a laparotomy in women with septate uterus is the possibility to evaluate and diagnose associated pelvic pathologies and to assess tubal patency [30]. However, this can easily be achieved by concomitant hysteroscopy and laparoscopy. For example, in our series, 12/57 women were found to have a uni- or bilateral tubal factor. In addition, concomitant laparoscopy helped us to control the septum dissection from outside the uterus, diagnose and manage uterine perforation, if it occurred, and to potentially minimize the risk of such a complication. On the other hand, however, most series included in this review did not use concomitant laparoscopy with no apparent disadvantage regarding safety and efficacy of the procedure.

We used a resectoscope for septoplasty in our series. In the investigated studies, several different methods and instruments for hysteroscopic septoplasty have been described, namely scissors, resectoscope, and argon laser. Data on the impact of the hysteroscopic technique on the reproductive outcome, however, are rare. Cararach et al. compared the reproductive outcome of patients operated by resectoscope with those in whom scissors have been used. They found a higher pregnancy rate in the scissors group [31]. On the other hand, Fedele et al. could not find a difference comparing the reproductive outcome results obtained with microscissors, argon laser, and resectoscope, in their series [7]. Thus, it is unclear, whether the use of a specific instrument may improve outcome and further comparative trials should be encouraged. Operative hysteroscopy may be performed using monopolar or bipolar electrosurgery. Bipolar electrosurgery uses isotonic saline as distension medium and may be safer and more effective for hysteroscopic surgery compared to monopolar electrosurgery based on one randomized trial [32]. A definitive recommendation, however, is premature at this time.

The insertion of intrauterine device (IUD) has been proposed as an option to potentially prevent both postoperative pregnancy and the development of intrauterine adhesions [30]. This practice, however, is not recommended and it's efficacy is unknown. In our series, we did not use an IUD system and advised the patients to use a safe contraceptive method for three months after surgery.

Re-operations were rarely necessary in our series and in the pooled analysis of all 18 trials with re-operation rates of 3% and 6%, respectively. This indicates that hysteroscopic uterine septoplasty can achieve effective removal of the septum in most cases. Adequate distension of the uterine cavity and loss of distension fluid through the cervix may influence the efficacy of septum removal and thus influence re-septoplasty rates. The influence of these factors, however, has not been investigated in the present study. Given that most series were published by public institutions with residency programs, these data also indicate that hysteroscopic uterine septoplasty may be considered an operation with a steep learning curve. This is consistent with our experience that this operation is easy to teach and easy to learn, especially with concomitant laparoscopy.

Another point of interest is whether or not a pre-operative therapy should be recommended prior to hysteroscopic uterine septoplasty. Preparation of the endometrium with various agents, including GnRH analogues, danazol, misoprostol, and PGE1 tablets, was used in 6/18 studies. This demonstrates that most surgeons do not find it necessary to use any form of pre-operative therapy. Furthermore, a recent randomized trial could not detect a benefit of 200 μg of sublingual misoprostol in women undergoing diagnostic hysteroscopy [33]. Also, the heterogeneity of the medications used does not allow to recommend a specific therapy.

It is of note that women undergoing hysteroscopic septoplasty are also at risk of uterine rupture in a subsequent pregnancy. Sentilhes et al. describe a total of 14 cases of uterine rupture after operative hysteroscopy in the literature [34]. Of these, 12 had a history of septoplasty. Although obviously a rare complication, patients should be made aware of this potentially serious late complication of hysteroscopic septoplasty.

## Conclusions

In summary, we describe a retrospective cohort study and a pooled analysis of 18 studies on the safety and efficacy of hysteroscopic uterine septoplasty in women with septate uterus. We found that this procedure is safe and effective with a subsequent pregnancy rate of 69% and a LBR of 49%.

## Competing interests

The authors declare that they have no competing interests.

## Authors' contributions

KN performed design, analysis and reporting of the study. JO performed acquisition of the data presented in Tables 2 and 3. LS and EF performed acquisition of the data presented in Tables 1, 2 and 3 and Figure 1. JCH organized the program. CBT drafted the manuscript and performed revisions of the manuscript. All authors read and approved the final manuscript.

## References

[B1] Grimbizis GF Camus M Tarlatzis BC Bontis JN Devroey P Clinical implications of uterine malformations and hysteroscopic treatment results Hum Reprod Update 2001 7 161 174 10.1093/humupd/7.2.161 11284660

[B2] Ashton D Amin HK Richart RM Neuwirth RS The incidence of asymptomatic uterine anomalies in women undergoing transcervical tubal sterilization Obstet Gynecol 1988 72 1 28 30 3380507

[B3] Acien P Acien M Evidence-based management of recurrent miscarriage. Surgical management Int Congr Series 2004 1 266 335 342 10.1016/j.ics.2004.02.003

[B4] Harger JH Archer DF Marchese SG Muracca-Clemens M Garver KL Etiology of recurrent pregnancy losses and outcome of subsequent pregnancies Obstet Gynecol 1983 62 5 574 581 6604890

[B5] Raga F Bauset C Remohi J Bonilla-Musoles F Simon C Pellicer A Reproductive impact of congenital uterine anomalies Hum Reprod 1997 12 2277 2281 10.1093/humrep/12.10.2277 9402295

[B6] Simon C Martinez L Pardo F Müllerian defects in women with normal reproductive outcome Fertil Steril 1991 56 1192 1193 1743344 10.1016/s0015-0282(16)54741-4

[B7] Fedele L Arcaini L Parazzini F Vercellini P Di Nola G Reproductive prognosis after hysteroscopic metroplasty in 102 women: life table analysis Fertil Steril 1993 59 768 772 8458494

[B8] Fayez J A comparison between abdominal and hysteroscopic metoplasty Obstet Gynecol 1986 68 3 399 403 2942813 10.1097/00006250-198609000-00023

[B9] Sparac V Kupesic S Ilijas M Zodan T Kurjak A Histologic architecture and vascularization of hysteroscopically excised intrauterine septa J Am Assoc Gynecol Laparosc 2001 8 1 111 116 10.1016/S1074-3804(05)60559-2 11172125

[B10] Dabirashrafi H Bahadori M Mohamd K Alavi M Mghadami Tabrizi N Zandinejad R Septate uterus: new ideas on the histological features on the septum in this abnormal uterus Am J Obstet Gynecol 1995 172 105 107 10.1016/0002-9378(95)90093-4 7847514

[B11] Mollo A De Franciscis P Colacurci N Cobellis L Perino A Venezia R Hysteroscopic resection of the septum improves the pregnancy rate of women with unexplained infertility: a prospective controlled trial Fertil Steril 2009 91 2628 2631 10.1016/j.fertnstert.2008.04.011 18571168

[B12] Hollett-Caines J Vilos GA Abu-Rafea B Ahmad R Fertility and pregnancy outcomes following hysteroscopic septum division J Obstet Gynaecol Can 2006 28 2 156 159 16643719 10.1016/s1701-2163(16)32069-2

[B13] Wang JH Xu KH Lin J Chen XZ Hysteroscopic septum resection of complete septate uterus with cervical duplication, sparing the double cervix in patients with recurrent spontaneous abortions or infertility Fertil Steril 2009 91 6 2643 2649 10.1016/j.fertnstert.2008.04.009 18565515

[B14] Valle RF Hysteroscopic treatment of partial and complete uterine septum Int J Fertil 1996 41 3 310 315 8799762

[B15] Ozgur K Isikoglu M Donmez L Oehninger S Is hysteroscopic correction of an incomplete uterine septum justified prior to IVF? Reprod Biomed Online 2007 14 3 335 340 10.1016/S1472-6483(10)60876-0 17359587

[B16] Venturoli S Colombo FM Vianello F Seracchioli R Possati G Paradisi R A study of hysteroscopic metroplasty in 141 women with a septate uterus Arch Gynecol Obstet 2002 266 3 157 159 10.1007/s004040100217 12197556

[B17] Doridot V Gervaise A Taylor S Frydman R Fernandez H Obstetric outcome after endoscopic transection of the uterine septum J Am Assoc Gynecol Laparosc 2003 10 2 271 275 10.1016/S1074-3804(05)60310-6 12732783

[B18] Jakiel G Robak-Chołubek D Przytuła-Piłat M Two-year study of women with fertility problems following uterine septum hysteroscopic treatment Ann Univ Mariae Curie Sklodowska 2004 59 2 65 69 16146051

[B19] Pace S Cipriano L Pace G Catania R Montanino G Septate uterus: reproductive outcome after hysteroscopic metroplasty Clin Exp Obstet Gynecol 2006 33 2 110 112 16903250

[B20] Colacurci N De Franciscis P Mollo A Litta P Perino A Cobellis L Small-diameter hysteroscopy with Versapoint versus resectoscopywith a unipolar knife for the treatment of septate uterus: a prospective randomized study J Minim Invasive Gynecol 2007 14 622 627 10.1016/j.jmig.2007.04.010 17848325

[B21] Colacurci N De Placido G Mollo A Carravetta C De Franciscis P Reproductive outcome after hysteroscopic metroplasty Eur J Obstet Gynecol Reprod Biol 1996 66 147 150 10.1016/0301-2115(96)02417-7 8735737

[B22] Saygili-Yilmaz ES Erman-Akar M Yilmaz Z A retrospective study on the reproductive outcome of the septate uterus corrected by hysteroscopic metroplasty Int J Gynecol Obstet 2002 78 59 60 10.1016/S0020-7292(02)00096-6 12113974

[B23] Pabuçcu R Gomel V Reproductive outcome after hysteroscopic metroplasty in women with septate uterus and otherwise unexplained infertility Fertil Steril 2004 81 1675 1678 10.1016/j.fertnstert.2003.10.035 15193494

[B24] Litta P Bonora M Pozzan C Merlin F Sacco G Fracas M Capobianco G Dessole S Carbon dioxide versus normal saline in outpatient hysteroscopy Hum Reprod 2003 18 2446 2449 10.1093/humrep/deg465 14585899

[B25] Marabini A Gubbini G Stagnozzi R Stefanetti M Filoni M Bovicelli A Hysteroscopic metroplasty Ann N Y Acad Sci 1994 734 488 492 10.1111/j.1749-6632.1994.tb21781.x 7978954

[B26] Kupesic S Kurjak A Diagnosis and treatment outcome of the septate uterus Croat Med J 1998 39 2 185 190 9575275

[B27] Porcu G Cravell L D'Ercole C Cohen D Roger V Blan B Hysteroscopic metroplasty for septate uterus and repetitive abortions: reproductive outcome Europ J Obstet Gynecol Reprod Biol 2000 88 81 84 10.1016/S0301-2115(99)00126-8 10659922

[B28] Guarino S Incandela S Maneschi M Vegna G D'Anna MR Leone S Maneschi F Hysteroscopic treatment of uterine septum Acta Eur Fertil 1989 20 5 321 325 2636810

[B29] Homer HA Li TC Cooke ID The septate uterus: a review of management and reproductive outcome Fertil Steril 2000 73 1 1 14 10.1016/S0015-0282(99)00480-X 10632403

[B30] Guarino S Incandela S Maneschi S Hysteroscopic treatment of uterine septum Acta Eur Fertil 1989 20 5 321 325 2636810

[B31] Cararach M Penella J Ubeda A Labatisda R Hysteroscopic incision of septate uterus: scissors versus resectoscope Hum Reprod 1994 9 87 89 8195357 10.1093/oxfordjournals.humrep.a138326

[B32] Garuti G Luerti M Hysteroscopic bipolar surgery: a valuable progress or a technique under investigation? Curr Opin Obstet Gynecol 2009 21 4 329 334 10.1097/GCO.0b013e32832e07ac 19512926

[B33] Mulayim B Celik NY Onalan G Bagis T Zeyneloglu HB Sublingual misoprostol for cervical ripening before diagnostic hysteroscopy in premenopausal women: A randomized, double blind, placebo-controlled trial Fertil Steril 2009 in press 10.1016/j.fertnstert.2009.01.073 19243750

[B34] Sentilhes L Sergent F Roman H Verspyck E Marpeau L Late complications of operative hysteroscopy: predicting patients at risk of uterine rupture during subsequent pregnancy Eur J Obstet Gynecol Reprod Biol 2005 120 2 134 138 10.1016/j.ejogrb.2004.10.010 15925040

